# Factors Associated with Medication Adherence among Community-Dwelling Older People with Frailty and Pre-Frailty in China

**DOI:** 10.3390/ijerph192316001

**Published:** 2022-11-30

**Authors:** Wenwen Cao, Chenglin Cao, Xin Zheng, Kai Ji, Qiming Liang, Yunwei Wu, Zhi Hu, Zhongliang Bai

**Affiliations:** School of Health Services Management, Anhui Medical University, Hefei 230032, China

**Keywords:** pre-frailty, frailty, medication adherence, older adults

## Abstract

Background: Frail and pre-frail older people often need to take medications. However, factors related to medication adherence among this population remain unclear, warranting further research. This study aims to identify correlates of medication adherence among frail and pre-frail older adults. Methods: From November 2020 to December 2020; a total of 4218 community-dwelling residents aged ≥ 60 years were interviewed by a cross-sectional survey in China. Data on subjects’ general information; medication adherence; and frailty status was obtained via the face-to-face structured questionnaire. Logistic regression models were fitted; separately; to examine these factors linked to medication adherence. Results: We found that 36.2% (*n* = 1527) and 18.8% (*n* = 792) of respondents were classified as pre-frail and frail. According to the Morisky scale scores, 66.74% (*n* = 2815) were found to have adequate medication adherence, and 33.26% (*n* = 1403) were found to have inadequate medication adherence. Among the pre-frail respondents, age (adjusted odds ratio (AOR) = 1.64; 95% confidence interval (CI): 1.18–2.29, *P* = 0.003), marital status (AOR = 1.52; 95% CI: 1.04–2.21, *P* = 0.030), smoking status (AOR = 0.61; 95% CI: 0.37–0.99, *P* = 0.044), and functional ability (AOR = 0.72; 95% CI: 0.58–0.91, *P* = 0.006) were significantly related to medication adherence. Among them, advanced age and single were risk factors, which were positively related to the medication adherence of subjects in pre-frailty, while quitting smoking and limited functional ability contributed to improving their medication adherence. In contrast, only age (AOR = 1.77; 95% CI: 1.16–2.69, *P* = 0.008) was significantly associated with medication adherence among frail subjects. Conclusion: Influencing factors to medication adherence of old people in pre-frailty and frailty have been enriched, which provides a certain reference for promoting medication adherence in this population. Future adherence intervention methods should be designed based on these factors.

## 1. Introduction

In recent years, with the acceleration of population aging taking place, many nations and regions have been facing unprecedented challenges [1,2], leading to increased concerns about the frailty of older adults. Frailty, characterized primarily as physical frailty, refers to a non-specific condition of reduced resilience to stress that could increase the incidence of unhealthy outcomes such as chronic falls, hospitalization, disability, and even death [3,4,5]. In addition, frailty seriously affects the daily activities of older adults, resulting in anxiety, depression, and other psychological problems, eventually compromising their quality of life [6,7]. Besides the abovementioned impacts of frailty on older people individually, frailty also imposes a great burden on the family and social development [8,9]. Accordingly, dealing with frailty properly and effectively has become an urgent public health concern. Indeed, prevention and management of frailty are significant in later life.

It is recognized that people have been found to take more than one medication due to comorbidities or other health issues in older age [10,11]. While results from a meta-analysis also found that comorbidities (two or more diseases) were very common problems amongst frail patients [12]. In practice, non-adherence or poor medication adherence often leads to treatment failure and increased healthcare costs [13]. Therefore, understanding factors related to medication adherence is especially important for health improvement and management among older people with frail or pre-frail status. 

In recent years, much emphasis has been placed on medication regimens among older people challenged by comorbidities such as diabetes, hypertension, and tumor [14,15,16,17,18,19]. Nonetheless, research that specifically determines barriers and facilitators to influence the development of medication adherence in frail or pre-frail older people, has been largely understudied.

Therefore, this study aimed to explore factors that are particularly associated with medication adherence among frail and pre-frail older people in the community setting. Findings of this paper may contribute to providing the foothold for promoting medication adherence and improving the quality of life and health management among these challenged communities.

## 2. Materials and Methods

### 2.1. Participants Recruitment and Data Collection

Data in this paper was from a cross-sectional study, which was investigated from November to December in 2020 via a multi-stage stratified random sampling method. In the first stage, four areas were selected according to their geographical location. Shanghai, Zhejiang Province, Jiangsu Province, and Anhui Province (Supplementary File S1). Next, two county-level regions (districts or counties including county-level cities) were randomly selected from each of the abovementioned areas, yielding a total of eight county-level regions. Then, one or two urban communities (streets) and rural communities (townships) were randomly selected from the abovementioned eight county-level regions. Finally, 24 neighborhood committees and villages were randomly selected as sampling sites.

According to the requirement, residents, who dwelled in the local community and were aged greater than or equal to 60 years, were invited for interview. To facilitate data collection, our investigators surveyed each subject via personally investigation using structured questionnaires. During this procedure, we did not take respondents who were incapable of verbal communication by reason of severe deafness or cognitive impairment, etc., into consideration. Before the interview, we verbally explained the purpose and steps of the investigation to participants in order to obtain their informed consent. In total, the present study enrolled 4257 subjects, after excluding invalid questionnaires, we included 4218 subjects for final analysis. 

### 2.2. Measures 

#### 2.2.1. Measurement of Frailty and Pre-Frailty 

In the present study, we used a measurement tool containing four dimensions and 23 items to assess the frail status of the respondents. We first calculated the scores of 23 items, then, we obtained the frail status score by dividing this score by 23. This study classified frailty into three categories; frailty scores < 0.12, 0.12–0.24, and ≥0.25 represented healthy (non-frail), pre-frail, and frail, respectively [20]. An excellent internal consistency was obtained according to the value for Cronbach’s alpha (Cronbach’s α = 0.771). For details of this measurement tool, the supplementary file can be referenced here (Supplementary File S2). 

#### 2.2.2. Measurement of Medication Adherence

Medication adherence, as the main dependent variable, was assessed with the four-item Morisky scale [21,22]. Respondents were asked to answer four questions regarding their medication regimes in daily life. In line with previous studies [23,24], participants were classified as having poor medication adherence if they selected “No” to any of the four questions; otherwise, they were considered to have good medication adherence. Based on the value for Cronbach’s alpha (Cronbach’s α = 0.502), we found that internal consistency was acceptable in this sample. A full description of the four-item Morisky scale has been placed here (Supplementary File S2). 

#### 2.2.3. Measurement of Demographic Data

During data collection, we obtained information on the characteristics of the subjects. These data consisted of age, gender, body mass index, residence, living status, marital status, and educational achievement. In addition, health behavior information as to smoking and drinking status, and social economic status regarding the source of income, medical insurance, and endowment insurance was also collected. 

Meanwhile, this study also collected information on self-reported diseases. For example, we used the Zung self-rating depression scale (SDS) (20 items) to assess depression, with options ranging from 1 to 4. During data analysis, depression scores were classified as no depression (<50), minimal to mild depression (50–59), and depression (>60), which was consistent with previous research [25,26,27]. We employed the 14 items composite scale developed by Lawton and Brody to determine the functional ability of the respondents [28]. Likewise, we also put the full text of these variables here (Supplementary File S2). 

### 2.3. Data Analysis

In the first step, we dichotomized medication adherence into two categories (adequate and inadequate medication adherence) [23,24] and expressed categorical variables with percentages and numbers. Then, the chi square test was performed to compare the differences between participants who were adequately adherent and inadequately adherent regarding the included variables. 

Next, to explore the factors related to medication adherence, this paper developed logistic regression models, separately. We initially performed univariate logistic regression models, then adjusted for potential covariates to obtain logistic regression models. The results yielded from these models were indicated with odds ratio (OR) and adjusted odds ratio (AOR) and corresponding 95% confidence interval (95% CI). All the results in this paper were generated by SPSS 23.0 statistical software and a *P* value smaller than 0.05 was deemed as statistically significant.

## 3. Results

### 3.1. Results of Descriptive Statistics

As presented in Table 1, participants classified as frail and pre-frail accounted for 18.8% and 36.2%, respectively. We found 33.26% (*n* = 1403) of the participants reported inadequate medication adherence, of which 43.0% (*n* = 603/1403) were between 70 and 79 years, with a female predominance (66.9%, *n* = 939/1403), living with others (88.7%, *n* = 1245/1403) and were married or cohabited with a partner (82.3%, *n* = 1155/1403). The source of income of half of the participants was from pensions (49.8%). Meanwhile, about two-thirds of participants (66.2%) had minimal to mild depression. Among participants with inadequate medication adherence, 42.3% (594/1403) were pre-frailty. Moreover, significant differences in many variables between the two groups (adequate versus inadequate medication adherence) were observed (*P* < 0.05). 

### 3.2. Factors Associated with Medication Adherence among Pre-Frail Subjects 

The results of the binary regression logistic analyses among pre-frail subjects are shown in Table 2. Bivariate logistic regression analyses (model 1) indicated that, in comparison with reference groups, participants aged 70 to 79 (OR = 1.79; 95% CI: 1.32–2.42) or greater than 80 years (OR = 1.43; 95% CI: 1.06–1.93), who lived alone (OR = 2.19; 95% CI: 1.59–3.01), and were single (OR = 1.98; 95% CI: 1.51–2.60), were at higher risk of inadequate medication adherence. Moreover, subjects who quit smoking (OR = 0.58; 95 % CI: 0.37–0.92) and had limited functional ability (OR = 0.77; 95 % CI: 0.63–0.95) were less likely to develop inadequate medication adherence. 

After adjustment, adjusted logistic regression results (model 2) revealed that, in comparison with the reference groups, respondents aged 70 to 79 (OR = 1.64; 95% CI: 1.18–2.29) who were single (OR = 1.52; 95% CI: 1.04–2.21) were at higher risk of inadequate medication adherence. Consistently, the interviewees who quit smoking (OR = 0.61; 95 % CI: 0.37–0.99) and had limited functional ability (OR = 0.72; 95 % CI: 0.58–0.91) were found to report better medication adherence. 

### 3.3. Factors Associated with Medication Adherence among Frail Subjects 

Unadjusted analyses (model 1) in Table 3 suggested that advanced age, such as 70 to 79 years (OR = 1.92; 95% CI: 1.31–2.82) and greater than 80 years (OR = 1.68; 95% CI: 1.17–2.42), living alone (OR = 1.51; 95% CI: 1.09–2.10), and being single (OR = 1.43; 95% CI: 1.06–1.92) were significantly associated with poor medication adherence. 

After controlling for variables, model 2 showed that only advanced age was significantly linked to an greater risk for poor medication adherence (70 to 79 years (OR = 1.77; 95% CI: 1.16–2.69) and greater than 80 years (OR = 1.67; 95% CI: 1.14–2.46)). 

## 4. Discussion

So far, this is the first study to explore factors related to medication adherence with a special focus on frail and pre-frail older adults. Overall, our results indicated that advanced age, marital status, smoking status, and functional ability were correlated with medication adherence amongst older adults who were pre-frail, while advanced age was found to have a significantly link among frail older adults. Over the years, few studies have examined factors associated with medication adherence with an emphasis placed on older people with frailty and pre-frailty, while age, gender, and education level have been related to medication adherence in patients who have diabetes, hypertension, and tumor [14,15,29]. The present study demonstrated that factors including gender, residence, and depressive symptoms were related to medication adherence for both groups, while these variables were not significantly related. In this regard, we found that older females were more likely to report adequate medication adherence, inconsistent with studies that reported that females had poor medication adherence, being busy taking care of family members and the house chores [30,31]. Currently, medication adherence studies have yielded inconsistent conclusions regarding gender differences, suggesting more research is warranted in the future. Furthermore, compared to urban-dwelling older people, counterparts from rural areas were inclined to have inadequate medication adherence despite being not significant, which was similar to a study that found that medication adherence was independent of the place of residence [32]. An increasing body of evidence suggests that older people with depression have poor medication adherence [10,33,34]. 

Herein, advanced age was associated with poor medication adherence among frail and pre-frail older people, which further indicated the impact of advanced age on health in later life, especially on medication adherence [35,36,37]. Indeed, poor medication adherence in later life may be explained by several reasons such as being incapable of handling a complex medication regimen, concerns about side effects, and forgetfulness [38,39]. In addition, our results revealed that medication adherence was relatively poor if participants lived alone among pre-frail older people, which substantiated that living alone was associated with inadequate medication adherence [40]. Similar to our results, older people with pre-frailty who were single had a higher risk of poor medication adherence, consistent with a prior study that concluded that married patients had better medication adherence than unmarried and widowed ones [41]. This finding may be explained by the fact that being married or cohabiting with a close partner could receive more care from their spouse/partner, who could remind them to take medication on time and help them to organize their medication [42]. 

Among examined factors in the present study, smoking status and functional ability were negatively correlated with medication adherence among older adults with pre-frailty. In other words, pre-frail older people who quit smoking and had limited functional ability were more likely to report better medication adherence. This finding may be attributed to the fact that smoking is well-established as an inducer of many diseases or conditions, and smokers are more likely to suffer from conditions that require them to take medication [43]. Moreover, a limited functional ability is related to unfavorable health-related outcomes, which may increase the need for drug treatment or therapy [44]. However, there is rich literature available substantiating that limited functional ability is associated with poor medication adherence among patients who are confronted with hypertension, chronic illness, and schizophrenia [10,45,46]. This finding may be explained by the fact that functional ability could only affect older people with pre-frailty. Indeed, to support our findings and better understand the role of functional ability in this community, more research is warranted. 

In this paper, the differences in factors related to medication adherence among pre-frail and frail older adults were observed. In other words, age, marital status, smoking status, and functional ability were associated with medication adherence among pre-frail older people. Specifically, older people who were of advanced age and single were more likely to report poor adherence, and those who quit smoking and had limited functional ability tended to report good adherence. While advanced age was only related to medication adherence among frail older people. A possible reason for this result could be explained by the average age of frail subjects (73.55 years) being older than pre-frail respondents (72.38 years). This finding further emphasized the impact of advanced age on health, especially medication adherence in later life, suggesting that more attention should be given to age when designing interventions aimed to improve medication adherence in the community.

Moreover, in the present study, we found that correlates of medication adherence among non-frail older adults were similar to pre-frail older adults based on our analysis (Supplementary File S3), which also indicated that marital status and functional ability were related to adherence. However, the role of body mass index had been observed in this population, indicating that older people who had underweight and normal weight were absent from poor medication adherence. This is hard to explain and more research is needed to verify this result.

Given that medication adherence determinants in frail and pre-frail older people have been underreported in the literature. How to design useful and meaningful interventions for addressing this concern has been seen as a priority for research and practice. The findings of our paper may play a role in understanding the determinants that affect adherence, thereby generating implications of great importance for designing tailored and targeted interventions to promote medication adherence in this population. For example, further effort is encouraged to be paid to older adults who are single and live alone. To this end, public health interventions aiming to educate this population on medication self-management should be introduced. Therefore, the intervention of such special influencing factors needs to start from two aspects. On the one hand, the family members of older people with limited functional ability should increase their care for them and remind them to take medicine on time. On the other hand, society and the government are encouraged to create friendly circumstances to help older adults with poor functional ability better adapt to society. This, eventually, may help them improve medication adherence.

However, several limitations were present in this study. The cross-sectional study design may not reflect the factors affecting medication adherence in older people who are frail or pre-frail. Therefore, a longitudinal study design is warranted in the future to validate the results found here. Furthermore, given that the data comes from the self-reporting of each respondent, there may be recall bias. Nevertheless, to minimize this bias and improve the data rightness, several solutions had been taken. For example, the composition of clear and precise questionnaire items and implementation of a pilot study before the investigation was introduced in our study. In addition, the use of the four-item Morisky scale medication adherence, which only measures the subjective attitude or cognition of the respondents to taking medication, suggests it is incapable of accurately reflecting the actual level of medication adherence in life. In future research, this limitation can be solved by a behavioral measure of adherence, such as objective surveillance of medication consumption data.

## 5. Conclusions

This study suggested that advanced age was connected with poor medication adherence among older adults with pre-frailty and frailty. Interestingly, marital status, smoking status, and functional ability were significantly related to medication adherence in older people with pre-frailty. In light of the findings of this paper, measures and programs should be designed to optimize medication management among community-dwelling people in later life.

## Figures and Tables

**Table 1:** Descriptive analysis results of participant characteristics (*n* = 4218).

Variables	Medication Adherence		r	*P* Value	Total *n* = 4218
	Adequate Adherence (*n* = 2815)	Inadequate Adherence (*n* = 1403)			
**Age (years)**					
60–69	1177 (41.8%)	602 (42.9%)	−0.027	0.076	1779 (42.2%)
70–79	1135 (40.3%)	603 (43.0%)			1738 (41.2%)
≥80	503 (17.9%)	198 (14.1%)			701 (16.6%)
**Gender**					
Male	1020 (36.2%)	464 (33.1%)	0.031	0.043	1484 (35.2%)
Female	1795 (63.8%)	939 (66.9%)			2734 (64.8%)
**BMI (kg/m^2^)**					
≤18.5	138 (4.9%)	72 (5.1%)	0.018	0.236	210 (5.0%)
18.5–22.9	940 (33.4%)	443 (31.6%)			1383 (32.8%)
23–27.4	1372 (48.7%)	687 (49.0%)			2059 (48.8%)
≥27.5	365 (13.0%)	201 (14.3%)			566 (13.4%)
**Residence**					
Urban	1572 (55.8%)	744 (53.0%)	0.027	0.084	2316 (54.9%)
Rural	1243 (44.2%)	659 (47.0%)			1902 (45.1%)
**Living status**					
Living alone	410 (14.6%)	158 (11.3%)	−0.046	0.003	568 (13.5%)
Living with others	2405 (85.4%)	1245 (88.7%)			3650 (86.5%)
**Marital status**					
Married/cohabited	2170 (77.1%)	1155 (82.3%)	−0.060	<0.001	3325 (78.8%)
Single	645 (22.9%)	248 (17.7%)			893 (21.2%)
**Education**					
Primary school and below	1700 (60.4%)	871 (62.1%)	−0.016	0.314	2571 (61.0%)
Junior school	636 (22.6%)	301 (21.5%)			937 (22.2%)
High school	361 (12.8%)	177 (12.6%)			538 (12.8%)
College and above	118 (4.2%)	54 (3.8%)			172 (4.1%)
**Smoking status**					
Smoking-quitter	194 (6.9%)	113 (8.1%)	0.017	0.260	307 (7.3%)
Smoker	415 (14.7%)	164 (11.7%)			579 (13.7%)
Non-smoker	2206 (78.4%)	1126 (80.3%)			3332 (79.0%)
**Drinking status**					
Drinking-quitter	122 (4.3%)	68 (4.8%)	0.023	0.143	190 (4.5%)
Drinker	453 (16.1%)	189 (13.5%)			642 (15.2%)
Non-drinker	2240 (79.6%)	1146 (81.7%)			3386 (80.3%)
**Income**					
Salary	213 (7.6%)	177 (12.6%)	0.013	0.399	390 (9.2%)
Pension	1646 (58.5%)	698 (49.8%)			2344 (55.6%)
Family providing	588 (20.9%)	256 (18.2%)			844 (20.0%)
Subsidy	247 (8.8%)	213 (15.2%)			460 (10.9%)
Others	121 (4.3%)	59 (4.2%)			180 (4.3%)
**Medicare insurance**					
Else	39 (1.4%)	50 (3.6%)	0.042	0.006	89 (2.1%)
Basic medical insurance system for urban employees	1315 (46.7%)	566 (40.3%)			1881 (44.6%)
Basic medical insurance for urban residents	582 (20.7%)	267 (19.0%)			849 (20.1%)
New rural basic medical insurance for rural residents	879 (31.2%)	520 (37.1%)			1399 (33.2%)
**Endowment insurance**					
Else	285 (10.1%)	285 (20.3%)	−0.070	<0.001	570 (13.5%)
Basic endowment insurance for the urban working group	1363 (48.4%)	584 (41.6%)			1947 (46.2%)
Social endowment insurance for non-working urban residents	427 (15.2%)	164 (11.7%)			591 (14.0%)
New rural social endowment insurance for rural residents	740 (26.3%)	370 (26.4%)			1110 (26.3%)
**Depression status**					
No depression	763 (27.1%)	446 (31.8%)	−0.041	0.008	1209 (28.7%)
Minimal to mild depression	2021 (71.8%)	929 (66.2%)			2950 (69.9%)
Depression	31 (1.1%)	28 (2.0%)			59 (1.4%)
**Frailty status**					
Robust	1495 (53.1%)	404 (28.8%)	0.248	<0.001	1899 (45.0%)
Pre-frailty	933 (33.1%)	594 (42.3%)			1527 (36.2%)
Frailty	387 (13.7%)	405 (28.9%)			792 (18.8%)

**Table 2:** Logistic regression analysis examining the correlation of medication adherence in pre-frail older adults (*n* = 1527).

Variables	Model 1		Model 2	
	OR, 95% CI	*P* Value	AOR, 95% CI	*P* Value
**Age (years)**				
60–69	-		-	
70–79	1.79, 1.32–2.42	<0.001	1.64, 1.18–2.29	0.003
≥80	1.43, 1.06–1.93	0.020	1.34, 0.97–1.84	0.071
**Gender**				
Male	-		-	
Female	0.87, 0.70–1.08	0.194	0.89, 0.66–1.19	0.437
**Body mass index (kg/m^2^)**				
18.5–22.9	-		-	
≤18.5	1.09, 0.78–1.53	0.603	1.18, 0.83–1.66	0.355
23–27.4	1.20, 0.71–2.05	0.496	1.28, 0.74–2.22	0.373
≥27.5	1.08, 0.79–1.48	0.642	1.06,0.77–1.47	0.716
**Residence**				
Urban	-		-	
Rural	1.00, 0.82–1.23	0.975	1.09, 0.85–1.40	0.489
**Living status**				
Living with others	-		-	
Living alone	2.19, 1.59–3.01	<0.001	1.46, 0.94–2.24	0.089
**Marital status**				
Married/cohabited	-		-	
Single	1.98, 1.51–2.60	<0.001	1.52, 1.04–2.21	0.030
**Education level**				
College and above	-		-	
Primary school and below	0.80, 0.44–1.45	0.460	0.93, 0.50–1.72	0.815
Junior school	0.85, 0.62–1.18	0.335	0.88, 0.61–1.26	0.493
High school	0.89, 0.62–1.28	0.537	0.89, 0.61–1.29	0.523
**Smoking status**				
Non-smoker	-		-	
Smoker	0.79, 0.54–1.15	0.217	0.75, 0.47–1.18	0.210
Smoking-quitter	0.58, 0.37–0.92	0.019	0.61, 0.37–0.99	0.044
**Drinking status**				
Non-drinker	-		-	
Drinker	0.91, 0.57–1.47	0.706	1.09, 0.63–1.87	0.758
Drinking-quitter	0.73, 0.43–1.23	0.237	0.97, 0.55–1.71	0.906
**Depression status**				
No depression	-		-	
Minimal to mild depression	0.81, 0.31–2.10	0.668	0.84, 0.32–2.21	0.716
Depression	0.79, 0.31–2.01	0.615	0.83, 0.32–2.15	0.695
**Functional ability**				
Well	-		-	
Limited	0.77, 0.63–0.95	0.013	0.72, 0.58–0.91	0.006

Note: Model 2 adjusted age, gender, body mass index, residence, living status, marital status, education level, smoking, drinking status, depression status, and functional ability; OR: odds ratio, AOR: adjusted odds ratio, 95% CI: 95% confidence interval; -: refers to reference group.

**Table 3:** Logistic regression analysis examining the correlation of medication adherence in frail older adults (*n* = 792).

Variables	Model 1		Model 2	
	OR, 95% CI	*P* Value	AOR, 95% CI	*P* Value
**Age (years)**				
60–69	-		-	
70–79	1.92, 1.31–2.82	0.001	1.77, 1.16–2.69	0.008
≥80	1.68, 1.17–2.42	0.005	1.67, 1.14–2.46	0.009
**Gender**				
Male	-		-	
Female	0.79, 0.58–1.07	0.132	0.87, 0.58–1.30	0.495
**Body mass index (kg/m^2^)**				
18.5–22.9	-		-	
≤18.5	0.83, 0.53–1.30	0.423	0.91, 0.57–1.45	0.703
23–27.4	0.84, 0.45–1.59	0.602	0.91, 0.47–1.76	0.781
≥27.5	0.90, 0.58–1.39	0.631	0.92, 0.59–1.45	0.721
**Residence**				
Urban	-		-	
Rural	0.98, 0.74–1.29	0.876	1.15, 0.82–0.61	0.408
**Living status**				
Living with others	-		-	
Living alone	1.51, 1.09–2.10	0.012	1.38, 0.88–2.16	0.155
**Marital status**				
Married/cohabited	-		-	
Single	1.43, 1.06–1.92	0.019	1.07, 0.70–1.64	0.757
**Education level**				
College and above	-		-	
Primary school and below	1.08, 0.43–2.75	0.866	1.10, 0.42–2.90	0.842
Junior school	0.99, 0.60–1.63	0.956	1.10, 0.64–1.90	0.724
High school	0.81, 0.45–1.45	0.472	0.89, 0.48–1.63	0.703
**Smoking status**				
Non-smoker	-		-	
Smoker	1.53, 0.92–2.54	0.103	1.73, 0.87–3.41	0.116
Smoking-quitter	1.36, 0.69–2.64	0.372	1.73, 0.79–3.77	0.167
**Drinking status**				
Non-drinker	-		-	
Drinker	1.00, 0.56–1.77	0.990	0.68, 0.33–1.39	0.290
Drinking-quitter	0.67, 0.31–1.41	0.289	0.52, 0.22–1.26	0.148
**Depression status**				
No depression	-		-	
Minimal to mild depression	0.88, 0.45–1.71	0.697	0.99, 0.50–1.96	0.979
Depression	1.52, 0.78–2.96	0.219	1.67, 0.84–3.29	0.142
**Functional ability**				
Well	-		-	
Limited	0.95, 0.70–1.28	0.719	0.87, 0.62–1.21	0.393

Note: Model 2 adjusted age, gender, body mass index, residence, living status, marital status, education level, smoking, drinking status, depression status, and functional ability. OR: odds ratio, AOR: adjusted odds ratio, 95% CI: 95% confidence interval; -: refers to the reference group.

## Data Availability

Not applicable.

## Supplementary Materials

The following supporting information can be downloaded at: https://www.mdpi.com/article/10.3390/ijerph192316001/s1, File S1: The location of sampling areas (Red areas); File S2: Measurement tools; File S3: Logistic regression analysis examining the correlation of medication adherence in robust older adults (*n* = 1899).
