# Supplementary material for: Factors Associated with Medication Adherence among Community-Dwelling Older People with Frailty and Pre-Frailty in China
Source: Int J Environ Res Public Health. 2022 Nov 30;19(23):16001. doi: 10.3390/ijerph192316001 (PMC9740801; doi:10.3390/ijerph192316001)
Supplement: Supplementary file 1 [file ijerph-19-16001-s001.zip › File S2.pdf]

Measurement Tools

Frailty Assessment Scale

1 Body weakness

1.1 Do you feel that your health is significantly worse than last year?

1. obvious 2. Less obvious 3. not obvious

1.2 Have you been eating a lot less recently?

1. obvious 2. Less obvious 3. not obvious

1.3 Have you lost any sudden, noticeable weight recently? (" Significant weight loss "means a weight loss of at least 6kg in the last six months or at least 3kg in the last one month)

1. obvious 2. Less obvious 3. not obvious

1.4 Do you have difficulty moving your limbs because of the inconvenience in your daily life?

1. Yes 2. Occasionally 3. No

1.5 Do you have difficulty maintaining your balance in your daily life?

1. Yes 2. Occasionally 3. No

1.6 Do you have a language barrier that makes your daily life difficult?

1. Yes 2. Occasionally 3. No

1.7 Do you have physical fatigue that makes your daily life difficult?

1. Yes 2. Occasionally 3. No

1.8 Do you often feel tightness in your chest?

1. Yes 2. Occasionally 3. No

1.9 Do you often feel physical pain? (pain in the limbs, head, neck, back, muscles, etc.)

1. Yes 2. Occasionally 3. No

2. Mental weakness

2.1 Have you been feeling down (unhappy) in the last month?

1. Yes 2. Occasionally 3. No

2.2 Do you have physical fatigue that makes your daily life difficult?

1. Yes 2. Occasionally 3. No

2.3 Have you been able to deal with the problems in your life in the last month?

1. able 2. Do not can 3. unable

2.4 Have you been able to enlighten yourself on the problems you have encountered in the last month?

1. able 2. Can sometimes 3. Unable

2.5 Overall, are you satisfied with your current life or job?

1. satisfaction 2. Sometimes satisfaction 3. dissatisfaction

3 Social Decline

3.1 Do you live alone?

1.yes 2.no

3.2 Do you often invite people to your home? (including friends, relatives, neighbours, etc.)

1.yes 2.sometime 3.no

3.3 Do you often visit other people's homes? (including friends, relatives, neighbours, etc.)

1.yes 2.sometime 3.no

3.4Do you think there are a lot of friends and relatives you can meet and confide in at any time? (including children, relatives, neighbours and friends)

1.more 2. not too much 3.few

3.5 Do you often feel lonely?

1.yes 2.sometime 3.no

Environmental weakness

4.1 Do you think your house is in bad condition?

1.bad 2.not too bad 3.not bad

4.2 Do you think your accommodation is not comfortable enough?

1.uncomfortable 2.not feel well 3.comfortable

4.3 Don't you like the surroundings of the house?

1.dislike 2.not too like 3.like

4.4 Do you think the transportation around your house is not easy?

1.yes 2.sometime 3.no

Morisky Medication Adherence Scale

1. Do you ever forget to take your medicine?

1, Yes 2. No

2. Are you careless at times about taking your medicine?

1, Yes 2. No

3. When you feel better, do you sometimes stop taking your medicine?

1, Yes 2. No

4. Sometimes if you feel worse when you take the medicine, do you stop taking it?

1, Yes 2. No

Zung Self-rating Depression Scale (SDS)

Questions	A Little Of The Time= 1	Some Of The Time= 2	Good Part Of The Time= 3	Most Of The Time= 4
1. I feel down hearted and blue.				
2. Morning is when I feel the best.				
3. I have crying spells or feel like it.				
4. I have trouble sleeping at night.				
5. I eat as much as I used to.				
6. I still enjoy sex.				
7. I notice that I am losing weight.				
8. I have trouble with constipation.				
9. My heart beats faster than usual.				
10. I get tired for no reason.				
11. My mind is as clear as it used to be.				
12. I find it easy to do the things I used to.				
13. I am restless and can’t keep still.				
14. I feel hopeful about the future.				
15. I am more irritable than usual.				
16. I find it easy to make decisions.				
17. I feel that I am useful and needed.				
18. My life is pretty full.				
19. I feel that others would be better off if I were dead.				
20. I still enjoy the things I used to do.				

Self-rated Functional Ability Status

1 Eating

① Independently Finish ② Partially Limited ③ Dependent

2 Bathing

① Independently Finish ② Partially Limited ③ Dependent

3 Grooming

① Independently Finish ② Partially Limited ③ Dependent

4 Clothing

① Independently Finish ② Partially Limited ③ Dependent

5 Toileting

① Independently Finish ② Partially Limited ③ Dependent

6 Indoor Activities

① Independently Finish ② Partially Limited ③ Dependent

7 Walking (about 50m)

① Independently Finish ② Partially Limited ③ Dependent

8 Ascending and Descending Stairs

① Independently Finish ② Partially Limited ③ Dependent

9 Using phone calls

① Independently Finish ② Partially Limited ③ Dependent

10 Shopping

① Independently Finish ② Partially Limited ③ Dependent

11 Doing laundry

① Independently Finish ② Partially Limited ③ Dependent

12 Using vehicles

① Independently Finish ② Partially Limited ③ Dependent

13 Taking Pills

① Independently Finish ② Partially Limited ③ Dependent

14 Saving or withdrawing money

① Independently Finish ② Partially Limited ③ Dependent
