# Supplementary material for: Factors Associated with Medication Adherence among Community-Dwelling Older People with Frailty and Pre-Frailty in China
Source: Int J Environ Res Public Health. 2022 Nov 30;19(23):16001. doi: 10.3390/ijerph192316001 (PMC9740801; doi:10.3390/ijerph192316001)
Supplement: Supplementary file 1 [file ijerph-19-16001-s001.zip › File S3.pdf]

Table S1. Logistic regression analysis examining the correlation of medication adherence in robust older adults (n = 1899)

Variables	Model 1		Model 2	
	OR, 95% CI	*P* value	AOR, 95% CI	*P* value
**Age (years)**				
60-69	-		-	
70-79	1.30, 0.89-1.89	0.172	1.22, 0.82-1.82	0.335
≥ 80	1.50, 1.03-2.20	0.035	1.42, 0.96-2.10	0.083
**Gender**				
Male	-		-	
Female	1.06, 0.85-1.33	0.606	1.07, 0.78-1.47	0.674
**Body mass index (kg/m^2)**				
18.5-22.9	-		-	
≦18.5	0.45, 0.22-0.93	0.030	0.42, 0.20-0.88	0.022
23-27.4	0.67, 0.47-0.95	0.023	0.68, 0.48-0.97	0.036
≧27.5	0.80, 0.58-1.10	0.173	0.80, 0.57-1.11	0.182
**Residence**				
Urban	-		-	
Rural	1.03, 0.82-1.29	0.820	1.04, 0.80-1.33	0.789
**Living status**				
Living with others	-		-	
Living alone	2.10, 1.25-3.52	0.005	1.52, 0.83-2.78	0.173
**Marital status**				
Married / cohabited	-		-	
Single	1.77, 1.26-2.49	0.001	1.51, 1.01-2.27	0.043
**Education level**				
College and above	-		-	
Primary school and below	0.96, 0.54-1.70	0.877	0.97, 0.54-1.77	0.931
Junior school	0.88, 0.64-1.21	0.426	0.80, 0.57-1.14	0.219
High school	1.03, 0.73-1.47	0.865	1.02, 0.71-1.46	0.919
**Smoking status**				
Non-smoker	-		-	
Smoker	0.81, 0.52-1.26	0.345	0.77, 0.45-1.33	0.348
Smoking-quitter	0.74, 0.45-1.24	0.255	0.64, 0.36-1.13	0.121
**Drinking status**				
Non-drinker	-		-	
Drinker	1.34, 0.69-2.58	0.390	1.72, 0.80-3.68	0.165
Drinking-quitter	1.51, 0.75-3.02	0.250	1.81, 0.84-3.91	0.129
**Depression status**				
No depression	-		-	
Minimal to mild depression	0.20, 0.02-2.21	0.188	0.27, 0.02-3.04	0.288

Depression	0.12, 0.01-1.31	0.082	0.17, 0.01-1.89	0.149
**Functional ability**				
Well	-		-	
Limited	0.65, 0.52-0.82	<0.001	0.60, 0.47-0.76	<0.001

Note: Model 2 adjusted age, gender, body mass index, residence, living status, marital status, education level, smoking, drinking status, depression status, functional ability.
OR: odds ratio, AOR: adjusted odds ratio, 95% CI: 95% confidence interval
-: refers to reference group
