# Supplementary figures and images for: Factors Associated with Medication Adherence among Community-Dwelling Older People with Frailty and Pre-Frailty in China
Source: Int J Environ Res Public Health. 2022 Nov 30;19(23):16001. doi: 10.3390/ijerph192316001 (PMC9740801; doi:10.3390/ijerph192316001)

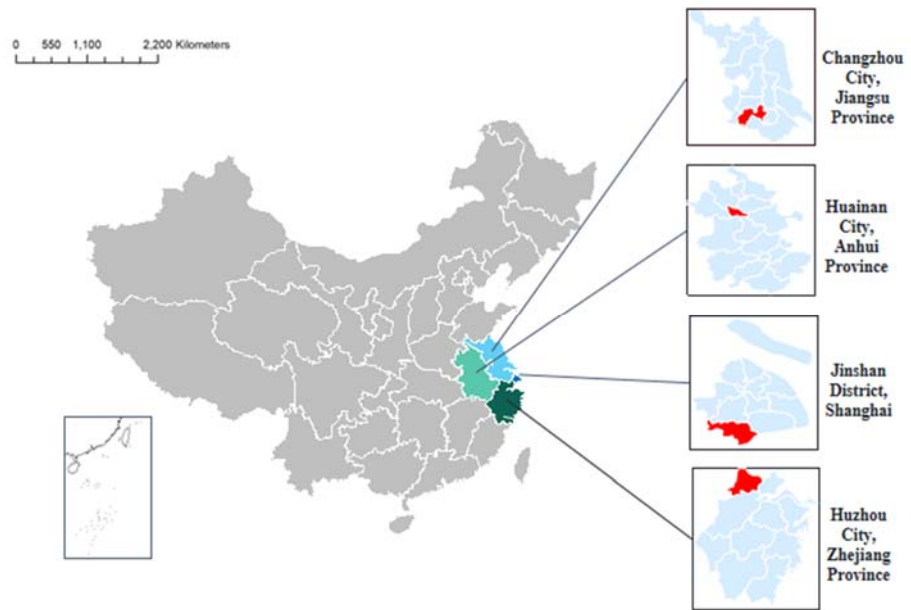

**Figure S1 . The location of sampling areas (Red areas).**

Supplement: Supplementary file 1 [file ijerph-19-16001-s001.zip › File S1.pdf]
